# Simultaneous Enhancement of Mechanical and Magnetic Properties in Extremely-Fine Nanograined Ni-P Alloys

**DOI:** 10.3390/nano8100792

**Published:** 2018-10-05

**Authors:** Qiongyao He, Wanquan Zhu, Xiaoxiao Fu, Ling Zhang, Guilin Wu, Xiaoxu Huang

**Affiliations:** 1 Joint International Laboratory for Light Alloys (Ministry of Education), College of Materials Science and Engineering, Chongqing University, Chongqing 400044, China; qyhe@cqu.edu.cn (Q.H.); wanquanzhu@cqu.edu.cn (W.Z.); zhangling2014@cqu.edu.cn (L.Z.); xiaoxuhuang@cqu.edu.cn (X.H.); 2 Department of Mechanical Engineering, Technical University of Denmark, DK-2800 Lyngby, Denmark

**Keywords:** nanocrystalline, magnetic, mechanical, grain boundary segregation, three-dimensional atom probe tomography (3D-APT)

## Abstract

Exploring structural effects that influence both the mechanics and magnetism in nanocrystalline materials, particularly extremely-fine nanograined ones with grain sizes down to several nanometers, is of high interest for developing multifunctional materials combining superior mechanical and magnetic performances. We found in this work that electrodeposited extremely-fine nanograined Ni-P alloys exhibit a significant enhancement of magnetization, simultaneously along with an increase in hardness, after low-temperature annealing. The relaxation of non-equilibrium structures, precipitation of the second phase and the segregation of P atoms to grain boundaries (GBs) during annealing have then been sequentially evidenced. By systematically comparing the variations in macroscopic and microstructural investigation results among several Ni-P alloys with different P contents, we suggest that the second phase has little effect on magnetization enhancement, and essentially both the structural relaxation and GB segregation can play important roles in hardening by governing GB stability, and in the improvement of magnetization by enhancing Ni–Ni atom exchange interactions.

## 1. Introduction

Nanocrystalline (NC) materials with grain sizes less than 100 nm normally exhibit excellent mechanical properties, such as high strength, high hardness and high wear resistance, which are significantly superior to their coarse-grained counterparts [1,2,3]. In parallel with mechanical properties, NC materials, such as Fe, Co, Ni and their alloys, also exhibit excellent soft or hard magnetic properties [4,5]. As developing NC materials combining both superior mechanical behaviors and favorable magnetic properties are of great practical interest for many applications, such as recording media and microelectromechanical systems/nanoelectromechanical systems [6,7], it is fundamentally important to explore some structural effects that are possibly able to influence the performance, in both mechanics and magnetism.

It is well known that the properties of NC materials are largely determined by the high volume of grain boundaries (GBs) in these materials and that these properties can be tuned by modifying GB structures. As for the mechanical properties optimization, the hardness of NC materials generally increases with decreasing grain size following the classical Hall-Petch relationship, but softening is observed when the grain size is smaller than 10–20 nm as the plastic deformation mechanism transfers from a dislocation-dominated process to a GB-mediated one [8,9,10,11,12]. In practice, NC materials can be further strengthened by lowering the GB energies through releasing non-equilibrium structures characterized by a high density of dislocations and misfit regions with low-temperature heat treatments [8,9,10,11,12]. Moreover, numerous computational models and experiments have suggested that segregation of solute atoms to GBs can sustain a stabilized nanostructure, thus providing the possibility to largely increase the strength in extremely-fine nanograined alloys with grain sizes below 10 nm [13,14,15,16,17,18,19,20]. As for the improvement of magnetic properties, present approaches mainly involve tailoring the chemistry and optimizing the microstructure [4,5]. Additive elements, such as P, Nb and Cu, can be added to NC magnetic alloys to achieve an expected magnetic performance with desired nanostructures [4,5,21]. However, few investigations provide nanostructural evidence of magnetic property changes related to GB structures in the case of extremely-fine nanograined alloys with grain sizes down to several nanometers. The possible influencing factors affecting both the improvement of the mechanical and magnetic properties are also rarely discussed.

In this study we explore the structural effects on both the strength and magnetization of extremely-fine nanograined metals by investigating Ni-P alloys. The NC Ni-P alloys, which generally have high strength and hardness [22], can also be used in magnetic devices, e.g., recording media [23]. Previous studies have shown that the magnetization in Ni-P alloys is mainly controlled by chemical composition [24,25,26], while the effect of P atoms in particular their distribution on magnetization is unclear and there still lacks detailed experimental characterizations on it. In the present work, transmission electron microscopy (TEM) and three-dimensional atom probe tomography (3D APT) techniques were used to study the role of P on hardness and magnetization of NC Ni-P alloys. Given that the solute P atoms may be segregated to GBs upon annealing due to the strong atomic attractive interaction [27], the possible effect of annealing-induced redistribution of P is of special concern in this work.

## 2. Materials and Methods 

The NC Ni-P alloys, with a thickness of ~60 μm, used in the present study were synthesized by direct current electrodeposition. The electrolyte was a citric acid-modified Watts bath containing 120 g/L nickel sulfate, 20 g/L nickel chloride, 15 g/L boric acid, and 15 g/L citric acid. All chemicals (purchased from Keshi-Chengdu Development Co., Ltd., Chengdu, China) were of analytical grade and used without further purification. Nickel sulfate and phosphorous acid were the sources of the Ni and P ions, respectively. The temperature of the bath was maintained at 70 °C with stirring, and the pH value was kept at 4 by adding sulfuric acid to the electrolyte. High purity Cu and Ni plates were used as the cathode and anode, respectively, for Ni-P alloy film electrodeposition. All coatings were deposited for a duration of 2 h to obtain a thickness of approximately 60 µm. The detailed processing methods can be found elsewhere [28]. Three materials were deposited and the contents of P in the alloys were determined to be 6.2 at.%, 7.1 at.% and 12.1 at.%, respectively, using energy dispersive spectroscopy. The as-deposited Ni-P alloys were then annealed at 325 °C for 0.5 h in an argon-protected atmosphere. Nanoindentation tests were performed at room temperature (RT) using a TI950 TriboIndenter (Hysitron, Minneapolis, MN, USA) with a standard Berkovich tip, following the Oliver-Pharr method [29] under a load-controlled mode with a peak load of ~4000 μN and a loading rate of 0.05 s^−1^. At least 30 indentations were performed in an area of 30 × 30 μm^2^ for each sample, and the load-displacement curves were obtained from the average values of 30 indentations, in order to eliminate the effect of the anisotropic mechanical behaviors on the indentation values. Magnetization curves of the as-deposited and as-annealed NC Ni-P alloys were measured using a vibrating sample magnetometer (VSM LakeShore 7404, Lake Shore Cryotronics Inc., Westerville, OH, USA) in the field between −1.2 MA/m ≤ H ≤ 1.2 MA/m at RT. The corresponding microstructures were characterized using a JEOL 2100 (JEOL Ltd., Tokyo, Japan) and FEI Tecnai G2 F20 (FEI, Hillsboro, OR, USA) TEM operated at 200 kV. The grain size was measured using dark field TEM images. At least 500 grains were counted for each sample. Atomic-scale compositional analyses were measured using a CAMECA LEAP 4000 HR 3D-APT (Cameca, Gennevilliers, France). 

## 3. Results and Discussions

**Figure 1:**
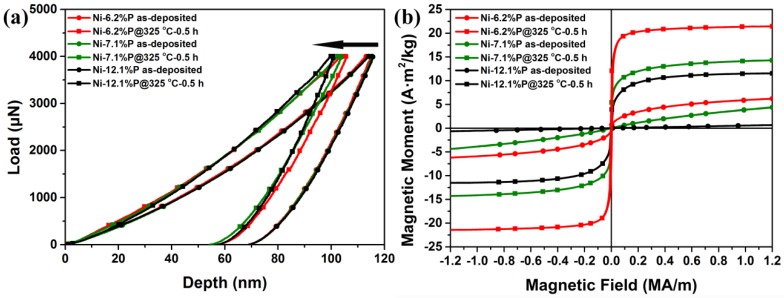
Typical displacement-load curves (**a**) and magnetic hysteresis loop curves (**b**) of as-deposited and 325 °C for 0.5 h annealed NC Ni-P alloys.

Figure 1a displays the typical nanoindentation displacement-load curves of the as-deposited and as-annealed NC Ni-P alloys. It is seen that the maximum penetration depths in the annealed Ni-P alloys were 55–60 nm, smaller than those in the as-deposited Ni-P alloys (~70 nm). The nanohardness of the annealed Ni-P alloys ranged from 9.80 ± 0.35 GPa to 10.37 ± 0.2 GPa, which was higher than that of the deposited Ni-P alloys (8.55–8.61 GPa). The corresponding RT magnetization curves are shown in Figure 1b. In the as-deposited case, it can be seen that the Ni-6.2%P alloy exhibited weak ferromagnetism, while Ni-7.1%P and Ni-12.1%P alloys exhibited a paramagnetic nature. The magnetization decreased from 6.22 A·m^2^/kg for Ni-6.2%P alloy to 0.76 A·m^2^/kg for Ni-12.1%P alloy under a magnetic field of 1.2 MA/m. After annealing at 325 °C for 0.5 h, all the Ni-P alloys showed a soft ferromagnetic nature and their saturation magnetizations increased significantly. For instance, the saturation magnetization of Ni-6.2%P alloy increased from 6.22 A·m^2^/kg to 21.53 A·m^2^/kg. Moreover, enhancements in both hardness and magnetization were found to be highly composition dependent in the Ni-P alloys, as the hardness increment after annealing increased with P concentrations, and the enhancement of magnetization decreased with increasing P concentrations.

**Figure 2:**
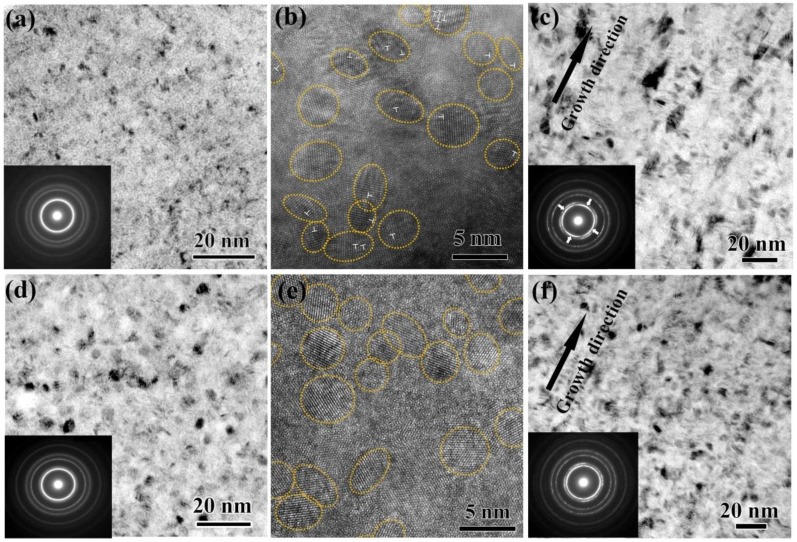
(**a**,**d**) planar bright field transmission electron microscope (TEM) images; (**b**,**e**) high resolution transmission electron microscopy (HRTEM) images and (**c**,**f**) bright field TEM images in growth direction of (**a**–**c**) as-deposited and (**d**–**f**) 325 °C for 0.5 h annealed Ni-7.1%P alloy, respectively. Dislocations were marked with white ‘T’. Insets are the corresponding selected area diffraction pattern (SADPs). The inhomogeneous distribution of reflection ring intensity in (c) was marked with white arrows.

The annealing-induced hardening and magnetization enhancement in the Ni-P alloys may be revealed from the corresponding microstructural evolution during annealing. The microstructures in the as-deposited and annealed Ni-7.1%P alloys are compared in Figure 2, and some small differences can be observed, both in the planar view and in the growth direction. As shown in Figure 2a, the as-deposited Ni-7.1%P alloy in the planar view is extremely fine, with grain sizes ranging from 1 nm to 3 nm, and it is a single-phase face-centered cubic (FCC) crystalline without diffraction spots from other phases, detected from its selected area diffraction pattern (SADP). After annealing at 325 °C for 0.5 h, the alloy remained a single-phase FCC crystalline, while GBs became sharper due to GB relaxation, and the grain sizes coarsened slightly to ~3.5 nm, as seen in Figure 2d. The high resolution transmission electron microscopy (HRTEM) images in Figure 2b,e reveal that the heavily distorted lattices, as well as the dislocations within nanograins in the as-deposited state, were eliminated after annealing, implying that these nanograins were free from defects after annealing. As seen in the growth direction, the columnar structures in Figure 2c, assembled by numerous nanograins and growth twins, were no longer obvious after annealing, as shown in Figure 2f. The strong texture in the as-deposited state turned out to be nearly random, since the reflection rings of the as-deposited state (the inset SADP of Figure 2c) appeared inhomogeneous in intensity while those of the as-annealing state (the inset SADP of Figure 2f) became relatively homogeneous. Very similar to Ni-7.1%P alloy, the as-deposited Ni-6.2%P and Ni-12.1%P alloys also had extremely-fine grains with grain sizes in the range of 1 nm to 3 nm, and minor changes, including slightly coarsened grains and eliminated defects, were observed in the microstructure after annealing as well, which is in agreement with previous literature [30].

Actual annealing-induced hardening in nanocrystalline metals has been reported to be due to the relaxation of non-equilibrium GBs in electrodeposited metals [15,22], and dislocation source strengthening in severe plastic deformed metals [31]. In essence, both structural relaxation processes can reduce the dislocation density and, accordingly, requires a higher flow stress to emit dislocations from the more stable GBs, resulting in an increased hardness. For the present Ni-P alloys, the hardening might be jointly contributed by the observed structural relaxation at GBs and in grain interiors. Meanwhile, structural relaxation can also contribute to magnetization enhancement. It has been accepted that relaxation-induced reduction in GB fraction and defect concentration, as presented in Figure 2, can lead to the reduction of disordered spin structures at the GB surface, as well as in the interior of grains, and thus increase saturation magnetization [32,33]. However, the increase is found to be highly composition-dependent, i.e., about two times for Ni-6.2%P alloy and 10 times for Ni-12.1%P alloy. Such a large difference cannot be explained by the very similar grain sizes among the three pairs of alloys, in both the as-deposited and as-annealing cases. Moreover, our alloys exhibited a much more significant increase compared with many other nanograined metals. For instance, normally, only up to a ~10% increase in saturation magnetization can be observed in Ni-Co electrodeposits after annealing, with reduced defects and doubled grain sizes [34]. Therefore, there should be significant additional contributions to magnetization enhancement in our alloys in addition to structural relaxation. 

**Figure 3:**
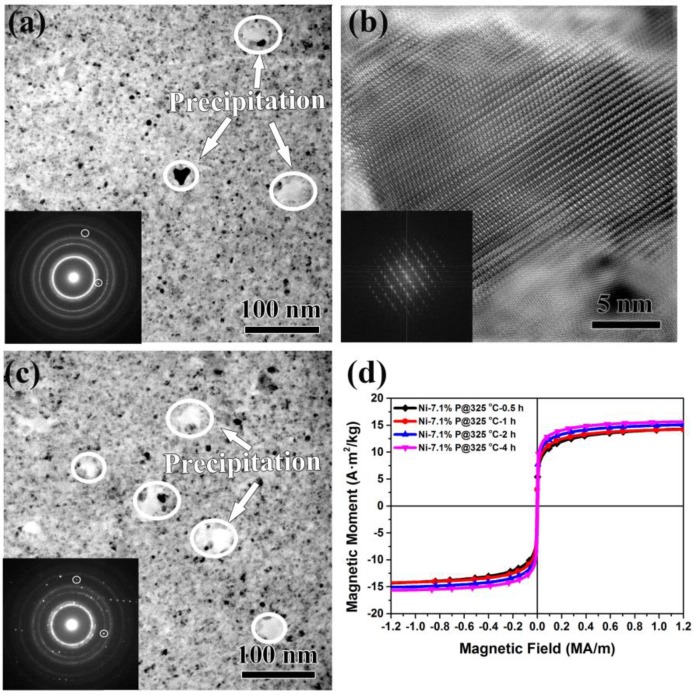
TEM images of Ni-7.1%P after annealing at 325 °C for (**a**) 2 h and (**c**) 4 h; (**b**) HRTEM of Ni_3_P. (**d**) The magnetic hysteresis loop curves of Ni-7.1%P annealed for different times. Insets are the corresponding SADPs. The white circles show the diffraction spots of Ni_3_P phase.

The crystallization of phases with higher magnetization upon annealing commonly plays an important role in the magnetization enhancement of magnetic alloys [4,5]. It has also been observed in amorphous Ni-P alloys that magnetization is enhanced due to the crystallization of FCC ferromagnetic phase Ni and paramagnetic phase Ni_3_P upon annealing [35]. However, the present alloys remained crystalline as single-phase FCC after annealing at 325 °C for 0.5 h, as mentioned above. Thus, the specific performance enhancement presented in Figure 1a,b may not be caused by phase transitions. To clarify the role of the second phase on the enhanced magnetization of our alloys, Figure 3 shows the microstructures and the corresponding magnetization curves of Ni-7.1%P alloy after annealing at 325 °C for 1–4 h. As shown in Figure 3a, second phases were precipitated after annealing at 325 °C for 2 h (volume fraction of ~3% with average grain size of ~20 ± 5 nm). The precipitations were indexed to be body-centered tetragonal structured Ni_3_P according to the HRTEM image in Figure 3b. Annealing for 4 h resulted in a larger volume fraction of Ni_3_P (volume fraction of ~15% with average grain size of ~40 ± 10 nm), as shown in Figure 3c. Although a large amount of Ni_3_P was precipitated after a longer annealing time, the saturation magnetization of Ni-7.1%P alloy was not significantly changed. The saturation magnetization was in agreement with the same P content after annealing at 400 °C with a large amount of Ni_3_P [36]. As revealed in Figure 3d, the saturation magnetization value only increased from 14.05 A·m^2^/kg after being annealed for 0.5 h, to 15.66 A·m^2^/kg after being annealed for 4 h, confirming that the second phase Ni_3_P had little effect on the improved saturation magnetization in the present NC Ni-P alloys. 

**Figure 4:**
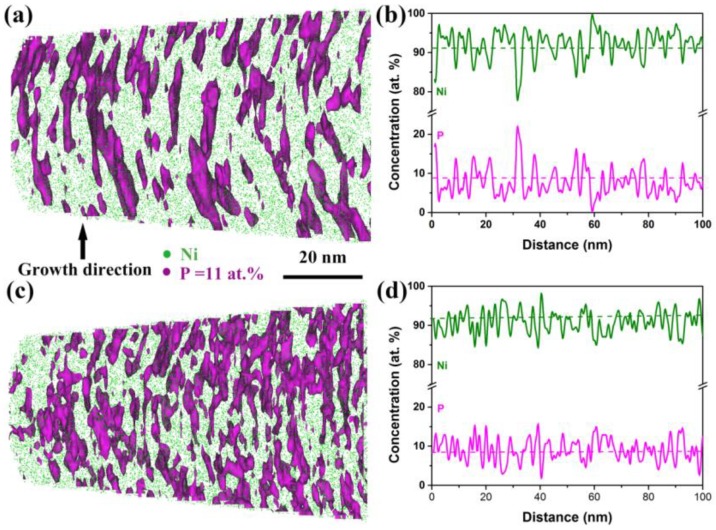
Three-dimensional atom probe tomography (3D-APT) analysis of solute elements distribution of (**a**) the as-deposited and (**c**) 325 °C for 0.5 h annealed Ni-7.1%P; (**b**) and (**d**) are the 1-D concentration profiles of the as-deposited and the annealed Ni-7.1%P, respectively.

Other than the second phase, the local chemical environment of as-deposited and annealed Ni-P alloys possibly carried more important information for exploring the origin of the properties’ optimization, since the hardness and magnetization enhancement of Ni-P alloys (shown in Figure 1) were observed to be composition dependent. A slice of a reconstructed 3D-APT map of the as-deposited Ni-7.1%P alloy with a thickness of 7 nm is shown in Figure 4a. The P surfaces with an iso-concentration of 11 at.% clearly shows that the solute P atoms were initially distributed heterogeneously in the NC Ni matrix. Some P atoms were soluble in the Ni matrix, whereas others were enriched along the growth direction. The 1D composition profile in Figure 4b shows several peaks with P concentrations above 11 at.%, and one of them even greater than 20 at.%, further confirming the inhomogeneous distribution of P. It can be seen in Figure 4c that the heterogeneous distribution of P atoms became more pronounced after annealing at 325 °C for 0.5 h. More P atoms were enriched along the growth direction, with a concentration above 11 at.%. The average distance of the peaks in Figure 4d is ~3 nm, corresponding quite well to the average grain size of Ni-7.1%P after annealing (Figure 2d–f). This demonstrates that more P atoms were segregated to GBs after annealing, which is in agreement with the studies of Hentschel et al. and Färber et al. [24,37].

The average P concentration within grains (*C*_g_) was then calculated using a simple mass balance, based on 3D-APT results, following the Equation [37,38]:

(1) $$C_{g}=\frac{C_{0}-f_{gb}C_{gb}}{f_{g}}$$

where *C*_0_ is the global P concentration and *C_gb_* is the P concentration in the GB regions. The *C*_0_ detected from the APT data before and after annealing were 9.09 at.% and 8.37 at.%, respectively, which were slightly higher than the nominalized concentration of the as-deposited state (7.1 at.%) due to the small composition variations in the as-deposited Ni-P alloys; *C_gb_* is assumed to be the average P concentration at the peak position in Figure 4b,d. The volume fraction of the grain interior (*f_g_*) and GB regions (*f_gb_*) were estimated using the following equation [33]:

(2) $$f_{g}=1-f_{gb}={\frac{\left(d-t\right)}{d^{3}}}^{3}$$

where *d* is the average grain size and *t* is the thickness of the GB (taken as 0.5 nm here) [34,35]. The P concentration in grain interiors before and after annealing for Ni-7.1%P alloy was calculated to be ~7.92 at.% and ~6.24 at.%, corresponding to 87% and 74% of the average concentrations, respectively, illustrating that the solute P atoms diffused from grain interiors to GBs during annealing and the remaining P concentration within grains was reduced after annealing.

The 3D APT results above evidenced that about 13% of the P atoms diffused from the interiors to GBs in Ni-7.1%P and, simultaneously, the P atoms at GBs redistributed at the boundary interfaces. The segregation of P to GBs actually plays important roles in enhancing the stability of GBs during annealing [15]. GB-mediated processes are suppressed in such conditions and a very high applied stress is required to generate extended partial dislocations from the stabilized GBs, which is particularly an issue for extremely-fine nanograined alloys, resulting in substantial hardening [15]. From this point of view, we can say that the Ni-6.2%P alloy had the smallest increase in hardening among the three alloys, because it contained the minimum amount of P, and, consequently, less P at the GBs suppressed the GB-mediated processes during annealing. On the contrary, the Ni-6.2%P alloy had the largest enhancement in magnetization in percentage terms. Taking the analysis of Figure 2 and Figure 3 into consideration, the enhancement difference among the three alloys is believed to be mainly caused by GB segregation. P is known to be a non-ferromagnetic element and P atoms are supersaturated solid solutions in the Ni matrix in as-deposited Ni-P alloys. P atoms take positions between nickel atoms and contribute electrons to fill the vacancies in the 3d shell of nickel atoms, making the Ni–Ni interaction change to a Ni–P–Ni interaction [39]. This decreases the exchange force between nickel atoms; therefore, the as-deposited Ni-P alloys exhibited paramagnetism and presented weak magnetization. After annealing, the concentration of P within the grains was decreased due to segregation of the P atoms to GBs, and the exchange interaction between nickel atoms in the grain interiors was enhanced, consequently resulting in an increased saturation magnetization. It is important to note that the maximum P concentration in the Ni GB regions is *C_gb_* = 15 at.% [24,35]. Comparing the Ni-P alloy with a low P concentration, i.e., Ni-6.2%P, the amount of P remaining in the grains increased after annealing the Ni-P alloy with a higher P concentration, i.e., Ni-12.1%P. The volume fraction of Ni–Ni exchange interaction within grains decreased; therefore, the saturation magnetization enhancement after annealing was less pronounced in the Ni-P alloys with a high P concentration. This may explain why the saturation magnetization enhancement in Ni–P alloys is highly composition-dependent. In addition, we understand that the hardness and the magnetization increase simultaneously with GB segregation, but their increase ratios do not necessarily follow a similar trend with the changes in the content of segregation elements. Future work for dealing with this issue may be interesting. 

## 4. Conclusions

In summary, a simultaneous enhancement of hardness and saturation magnetization during annealing was observed in extremely-fine nanograined Ni-P alloys with grain sizes down to 1–3 nanometers. To explore the structural origin, corresponding microstructures were systematically investigated via TEM and 3D-APT. It was evidenced that a reduction in defect concentration and GB fraction due to structural relaxation, the precipitation of a second phase, and the segregation of P atoms to GBs occurred during annealing. It is clarified that the commonly suggested precipitation of a second phase Ni_3_P has little effect on magnetization, while both the non-equilibrium structural relaxation and GB segregation played important roles in hardening by governing GB stability, and in the magnetization improvements by enhancing Ni–Ni atom exchange interactions. Additionally, the effects of GB segregation on the enhancement ratio of hardness and magnetization may follow opposite trends to the changes in content of the segregation elements.
